# The atypical E2F transcription factor DEL1 modulates growth–defense tradeoffs of host plants during root-knot nematode infection

**DOI:** 10.1038/s41598-020-65733-3

**Published:** 2020-06-01

**Authors:** Satoru Nakagami, Kentaro Saeki, Kei Toda, Takashi Ishida, Shinichiro Sawa

**Affiliations:** 1 Graduate School of Science and Technology, Kumamoto University, Kumamoto, 860-8555 Japan; 2 International Research Organization for Advanced Science and Technology (IROAST), Kumamoto University, Kumamoto, 860-8555 Japan

**Keywords:** Plant sciences, Plant immunity, Microbe

## Abstract

In plants, growth–defense tradeoffs are essential for optimizing plant performance and adaptation under stress conditions, such as pathogen attack. Root-knot nematodes (RKNs) cause severe economic losses in many crops worldwide, although little is known about the mechanisms that control plant growth and defense responses during nematode attack. Upon investigation of *Arabidopsis thaliana* infected with RKN (*Meloidogyne incognita*), we observed that the atypical transcription factor *DP-E2F-like 1* (*DEL1*) repressed salicylic acid (SA) accumulation in RKN-induced galls. The *DEL1*-deficient Arabidopsis mutant (*del1-1*) exhibited excessive SA accumulation in galls and is more resistant to RKN infection. In addition, excessive lignification was observed in galls of *del1-1*. On the other hand, the root growth of *del1-1* is reduced after RKN infection. Taken together, these findings suggest that *DEL1* plays an important role in the balance between plant growth and defense responses to RKN infection by controlling SA accumulation and lignification.

## Introduction

Salicylic acid (SA) is a key plant defense hormone required for immunity against pathogen infection^1^. Pathogens often induce SA accumulation both locally and systemically. This pathogen-induced SA is synthesized through the isochorismate pathway comprised of ISOCHORISMATE SYNTHASE 1 (ICS1), ENHANCED DISEASE SUSCEPTIBILITY 5 (EDS5), and avrPphB SUSCEPTIBLE 3 (PBS3)^2^. A recent study has revealed that ICS1, EDS5 and PBS3 are necessary for pathogen-induced SA biosynthesis in plants^2^. SA accumulation leads to the activation of plant defense responses, including programmed cell death and systemic acquired resistance. High levels of SA accumulation result in changes in transcriptional activities of genes with antimicrobial activity such as the *PATHOGENESIS-RELATED* (*PR*) genes^3^. In *Arabidopsis thaliana*, SA-dependent transcriptional reprogramming requires NONEXPRESSER OF PR GENES 1 (NPR1), which was shown to be the *bona fide* SA receptor and also functions as a transcriptional co-activator^4^. Many studies have reported SA-induced defense responses upon infection by leaf-colonizing pathogens^5^, while little is known about the involvement of SA-induced defense responses in roots.

Several studies have shown that SA metabolism is also targeted by phytoparasitic root-knot nematodes (RKNs; *Meloidogyne* spp.) to ensure successful infection^6^. RKNs cause severe yield losses of many crop species in multiple regions of the world^7^. During RKN infections, juveniles invade plant roots and move toward the vascular cylinder. Upon reaching the vascular cylinder, RKNs induce the formation of galls, each containing several giant cells (GCs) which serve as a source of nutrients for the nematode until maturity. Since GCs result from nuclear divisions without cytokinesis (endoreduplication), it is thought that RKN is capable of manipulating the host cell-cycle machinery^6^. Biotrophic pathogens such as RKNs must keep the host cells alive while also suppressing host defense mechanisms. Repression of genes of the SA pathway was indeed observed in RKN-induced galls^8–10^. In addition, overexpression of *NPR1* leads to the decrease in the number of galls and RKN fecundity during RKN infection^11^. On the other hand, overexpression of SA-degrading salicylate hydroxylase leads to reduced SA accumulation and greater susceptibility to RKN^12^.

SA accumulation contributes to plant defense responses, while excessive SA often leads to growth inhibition. Therefore, it is generally believed that host plants must balance growth and defense during pathogen infection. How this is accomplished is currently unknown. The Arabidopsis atypical transcription factor *DP-E2F-like 1* (*DEL1*)/*E2Fe*, a transcriptional repressor known to promote the onset of endoreduplication, has been shown to balance plant growth and defense via the SA response in leaves during pathogenic fungi infection^13^. Here, we report that DEL1 represses excessive SA accumulation and root growth inhibition of host plants upon RKN infection. Furthermore, lignin deposition was also shown to be elevated in galls of the *del1-1* knockout mutant. Our results suggest that *DEL1* plays a role in balancing growth and defense in roots as previously reported in leaves^13^.

## Results

### SA biosynthesis is enhanced in *del1-1* galls

**Figure 1:**
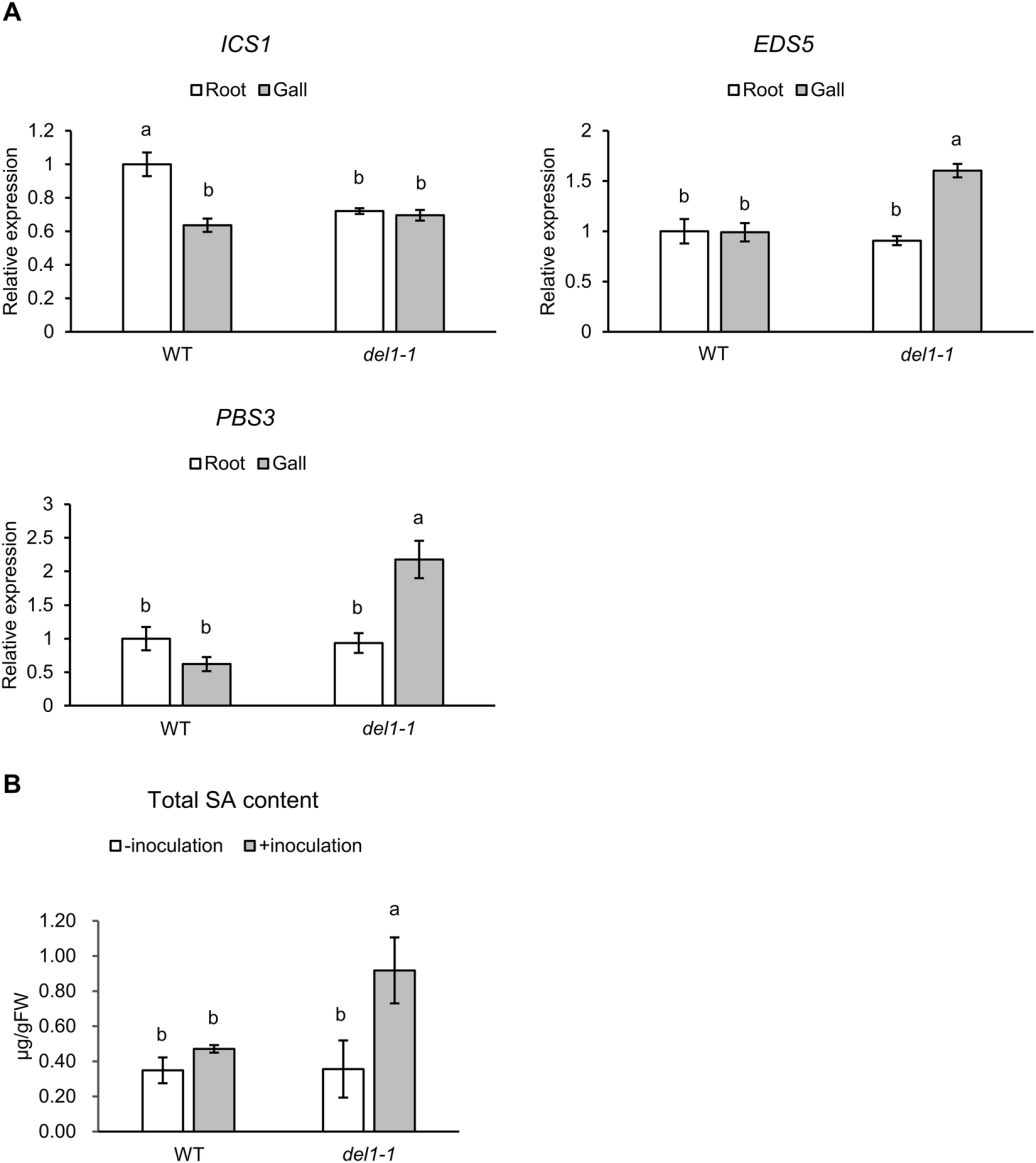
*DEL1* regulates SA biosynthesis during RKN infection. (**A**) RT-qPCR analysis of SA biosynthesis-related genes in WT and *del1-1* galls at 7dpi. Values are normalized to the expression levels in un-inoculated roots of WT. The experiment was repeated three times with similar results. (**B**) Quantification of free and total SA in the WT and the *del1-1* mutant. The experiment was repeated three times with similar results. Means ± SD are shown. Different letters denote significant differences after Tukey’s multiple test at *P* < 0.05.

Chandran *et al*. showed that *DEL1* acts as a transcriptional repressor of *EDS5*^13^. The enhanced resistance to fungi and the small stature phenotypes of *del1-1* were shown to be SA-dependent. *DEL1*-mediated SA accumulation has been demonstrated in leaves^13^, but it is unclear whether plants also balance growth–defense tradeoffs in the roots. To address this question, we subjected *del1-1* to the RKN infection assay. We performed reverse transcription quantitative polymerase chain reaction (RT-qPCR) on genes related to SA biosynthesis in wild-type (WT) and *del1-1* galls. *EDS5* and *PBS3* were up-regulated in the galls of *del1-1* compared to the WT, while *ICS1* did not show significant difference between transcript levels in *del1-1* and WT galls (Fig. 1A). We next tested whether SA accumulation in *del1-1* galls was higher than that in WT galls. Indeed, total SA accumulation in *del1-1* galls was significantly higher than that in WT galls (Fig. 1B). On the other hand, total SA level in un-inoculated roots of *del1-1* did not change significantly compared to that of WT (Fig. 1B). These results showed that *DEL1* represses SA biosynthesis in RKN-induced galls.

### Gall formation is compromised in *del1-1*

**Figure 2:**
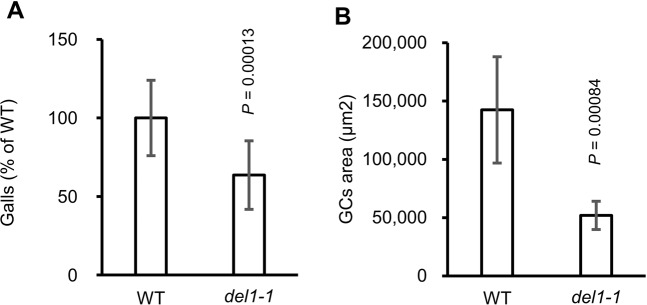
Gall formation is compromised in *del1-1* mutants. (**A**) WT-normalized gall numbers in WT and *del1-1* plants at 7 dpi (*n* = 15, WT; *n* = 16, *del1-1*). Sample sizes denote the number of petri dishes were tested. (**B**) Areas of GCs of 14 dpi galls in WT and *del1-1* plants (*n* = 6). Sample sizes denote the number of galls. Means ± SD are shown. *P* values were analyzed by Student’s *t*-test.

To determine whether the elevated SA levels activate the defense response, we analyzed the expression of the SA-dependent genes *PR1*, *PR2*, *PR5* and *NPR1* in galls. *PR1* transcripts were not detected from the galls of neither WT nor *del1-1* (Fig. S1), suggesting that *PR1* is likely not involved in RKN infection. On the other hand, *PR2* and *PR5* were up-regulated in the galls of *del1-1* compared to the WT, whereas *NPR1* expression level did not change (Fig. S1). These results suggest transcript level of *PR2* and *PR5* may be modified by SA accumulation in the galls of *del1-1*. Previous studies have shown that SA signaling confers increased host plant resistance to nematode infection^11,12,14,15^. To examine the functions of *DEL1* in RKN-induced gall formation, we assayed gall formation frequencies and GC formation in *del1-1*. Gall numbers were reduced in the *del1-1* mutant compared to that in the WT (Fig. 2A). Furthermore, histological analysis showed that GC areas were significantly reduced in *del1-1* at 14 days post inoculation (dpi) by approximately one third of the WT value (Figs. 2B and S2), which is consistent with the results from a previous study^16^. These results suggest that excessive SA accumulation from the loss-of-function of *del1-1* mutation renders the host plant more resistant to RKN infection. Therefore it is possible that the endocycle machinery itself is involved in the RKN infection together with SA signaling, as de Almeida Engler *et al*. (2019) has shown that *del1-1* mutant with defect of the endocycle machinery produced malformed giant cells^16^.

### *DEL1* is involved in lignin deposition during RKN infection

**Figure 3:**
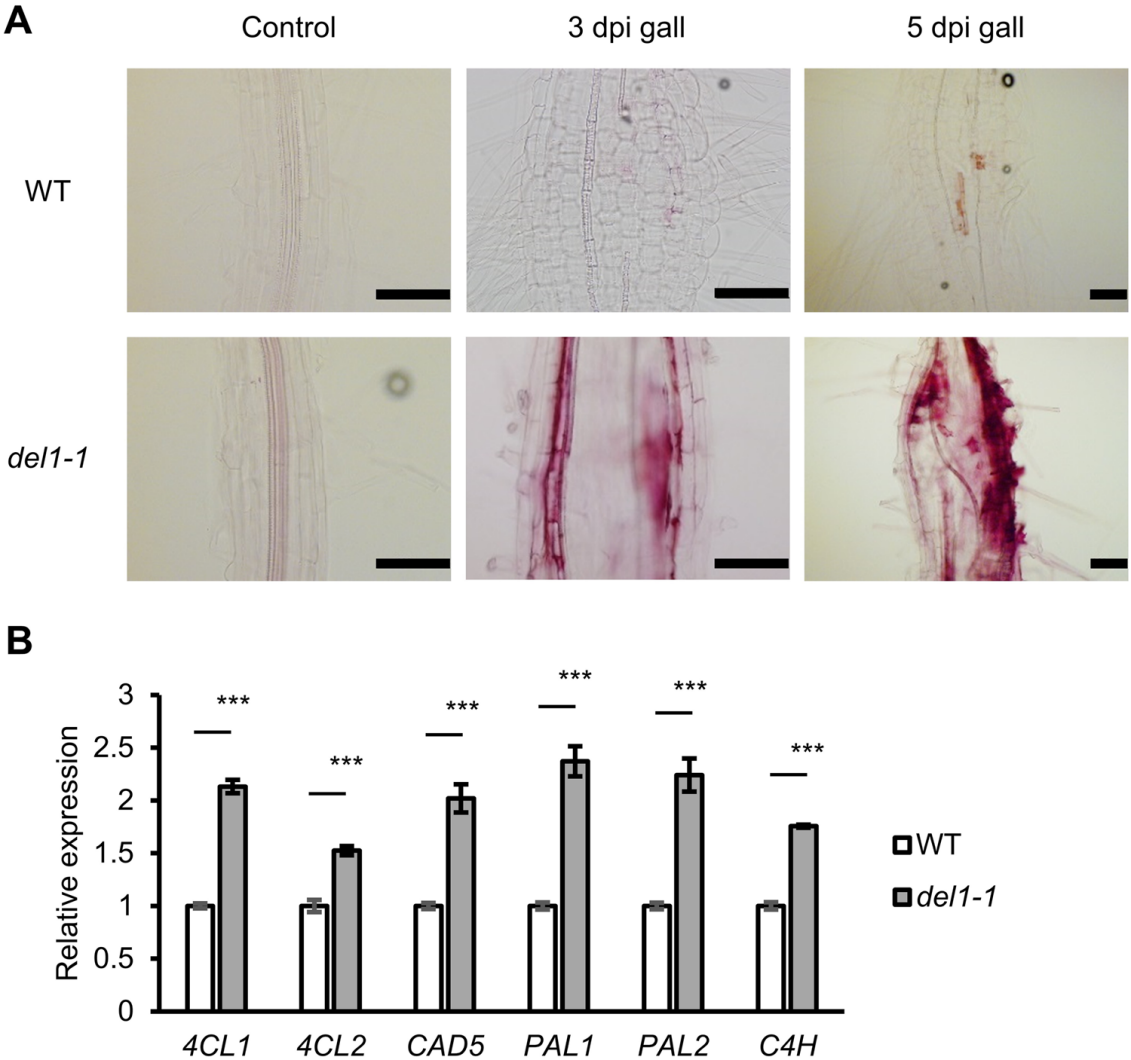
Lignin accumulates specifically in *del1-1* plants after RKN infection. (**A**) Phloroglucinol-HCl staining in non-infected regions of RKN-infected roots (control), 3 dpi and 5 dpi galls (5 individuals were observed with similar results). Top: WT. Bottom: *del1-1*. Scale bars = 100 µm. (**B**) RT-qPCR analysis of lignin synthesis-related genes in WT and *del1-1* 7 dpi galls. Values are normalized to expression levels in the WT. 4CL: 4-coumarate-CoA ligase, CAD: cinnamyl alcohol dehydrogenase, PAL: phenylalanine ammonia-lyase, C4H: cinnamate 4-hydroxylase. The experiment was repeated three times with similar results. Means ± SD are shown. ***P < 0.001 by Student’s *t*-test.

In Arabidopsis roots, cell wall lignification is restricted to the xylem and the Casparian strip during normal development^17^. Lignin contributes to the protection of plants from physical and chemical stresses, including pathogen challenges. Several studies have revealed that lignification can affect parasitic nematode infection rates^18–21^. Galls during early stages were subjected to phloroglucinol-HCl staining to detect lignin, which was weakly stained in 3 dpi galls but became more prominent in 5 dpi galls in WT (Fig. 3A). In contrast, lignin was strongly stained in both 3 dpi and 5 dpi galls of *del1-1* (Fig. 3A). In fact, 5 dpi galls of *del1-1* showed stronger lignin staining than WT galls at 5 dpi (Fig. 3A). Non-galling parts of RKN-infected seedlings in both WT and *del1-1* plants did not show lignin staining except for the vascular tissues (Fig. 3A). Next, we analyzed the transcript levels of lignin biosynthesis-related genes in galls by RT-qPCR. The expression levels of *4-coumarate: CoA ligase 1* (*4CL1*), *4CL2*, *alcohol dehydrogenase 5* (*CAD5*), *phenylalanine ammonia-lyase 1* (*PAL1*), *PAL2*, and *cinnamate 4-hydroxylase* (*C4H*) were significantly up-regulated in *del1-1* galls compared to WT galls (Fig. 3B). These results indicate that *DEL1* negatively regulates local lignin deposition during RKN infection.

### *del1-1* exhibits root growth inhibition after RKN infection

**Figure 4:**
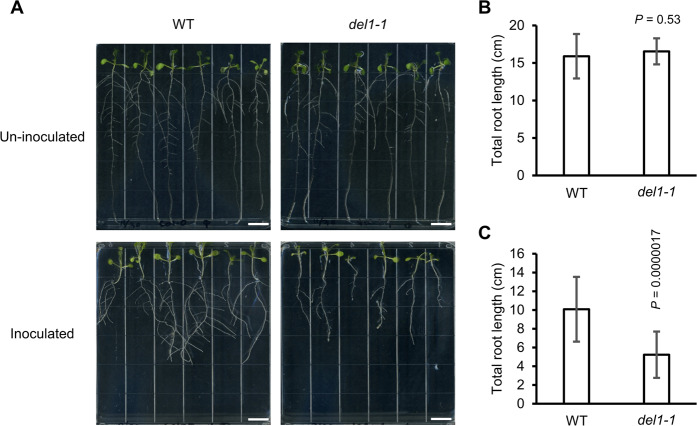
Root growth is inhibited in *del1-1* mutants after RKN infection. (**A**) Representative images of WT and *del1-1* seedlings with or without RKN inoculation. Top: 10-days-old non-inoculated seedlings. Bottom: RKN-inoculated seedlings at 7 dpi. (**B**) Total root length (primary root + lateral roots) of non-inoculated WT and *del1-1* plants (*n* = 12, WT; *n* = 11, *del1-1*). (**C**) Total root length of RKN-inoculated WT and *del1-1* plants (*n* = 18). Means ± SD are shown. Sample sizes denote the number of plants. The experiment was repeated three times with similar results. *P* values were analyzed by Student’s *t*-test.

Chandran *et al*. (2014) showed that *DEL1* controls rosette leaf size upon pathogen infection, while SA accumulation and lignin deposition are often associated with inhibition of growth in *Arabidopsis*^13,22–27^. We therefore questioned whether *DEL1* controls the balance between root growth and immunity after RKN infection. 10-day-old un-inoculated *del1-1* seedlings did not show significant differences in root growth compared to WT (Fig. 4A,B), whereas the total root length of RKN-inoculated *del1-1* seedlings at 7 dpi was significantly reduced compared to WT (Fig. 4A,C). These results indicate that *DEL1* plays an important role in the balancing growth–defense tradeoffs in root during RKN-infection (Figs. 2 and 4).

## Discussion

**Figure 5:**
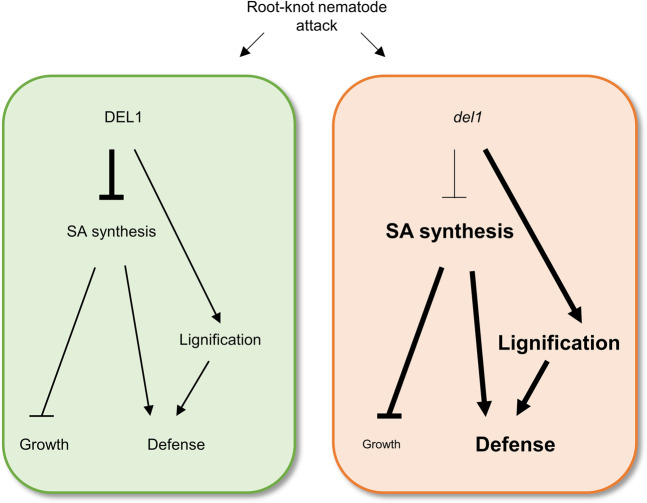
Schematic model depicting the role of *DEL1* during RKN infection. Proposed role for *DEL1* as a mediator plant growth and defense in the RKN infection in Arabidopsis roots. In WT plants, *DEL1* controls basal SA revels and lignification in galls after RKN attack to balance plant growth and defense (green panel). Excessive SA accumulation and lignification due to misexpression of *DEL1* contribute to plant defense after RKN attack, whereas the root growth is inhibited (orange panel).

In plants, the activation of immunity is often inversely correlated with growth. Thus, the tradeoff between immunity and growth is thought to be important for survival of plants under biotic stresses. We demonstrated that knocking out *DEL1/E2Fe* leads to enhanced resistance and growth inhibition during RKN infection, likely due to excessive lignification and/or SA accumulation in RKN-induced galls (Fig. 5). Our RT-qPCR analysis showed that *PR2* and *PR5* were up-regulated in *del1-1* galls, but *PR1* was not (Fig. S1). This suggests a portion of the SA metabolism genes respond to RKN-induced SA accumulation in *del1-1* galls. Since Fu *et al*. (2012) has shown that NPR1 protein level is up-regulated post-transcriptionally after SA treatment^28^, NPR1 proteins thus are likely to be highly accumulated in *del1-1* galls (Fig. S1). A number of studies have revealed that alterations in lignin biosynthesis result in changes in both growth and defense, while excessive lignification inhibits plant growth^25–27,29^. In addition, transgenic and mutant plants with elevated basal SA levels also exhibit growth reduction^13,22–24^. Moreover, recent studies have shown that SA accumulation in response to pathogen attack positively correlates with lignin deposition and acquisition of immunity^30–33^. In light of these lines of evidence, DEL1 may mediate the balance between defense and growth by limiting SA accumulation during RKN infection in root. Chandran *et al*. (2014) have reported that *del1-1* plants exhibit small rosette leaves and SA accumulation higher than that of WT regardless of fungal infection^13^. On the other hand, our results showed that *del1-1* exhibited root growth inhibition only in the presence of RKN infection (Fig. 4). SA accumulation in roots of *del1-1* in the absence of RKN was not significantly different compared to that of WT (Fig. 1B), suggesting this difference in response between in leaves and in roots may come from differences of SA level in the absence of pathogen infection.

Although reduced parasitic nematode fecundity on *del1-1* has been attributed to the deficiency in cell division during syncytia/gall formation^16^, our results point to the additional possibility that the up-regulation of SA accumulation and lignification may also be contributing factors in gall formation efficiency. Further detailed analysis of the function of DEL1 in SA accumulation/lignification during RKN infection may provide insights into the mechanisms of balancing defense and growth in plants.

## Methods

### Plant materials and growth conditions

All *Arabidopsis thaliana* plants used in this study are of the Col-0 ecotype background. The mutant line, *del1-1*, was obtained from the Arabidopsis Biological Resource Center (ABRC). Arabidopsis seeds were stratified for 2 days in 4 °C in the dark, then allowed to germinate and grown for 5 days on 0.25 × Murashige and Skoog (MS) salt mixture (Sigma), 0.5% (w/v) sucrose, and 0.6% (w/v) gellan gum at pH 6.4 under continuous light at 23 °C.

### Nematode preparation and inoculation

Root-knot nematodes (RKN, *Meloidogyne incognita*) were prepared aseptically as described previously^34^. Briefly, 6- to 7-week-old tomato plants were inoculated at 3-day intervals for a total of four inoculations. Approximately 80,000 juveniles were used to inoculate each plant. The inoculated tomato plants were then transferred to a hydroponic system, and after 2 to 4 day intervals infective juveniles were collected from the hydroponic culture media. Six Arabidopsis seeds were sown on a MS plate. Five-days-old Arabidopsis seedlings were inoculated with approximately 80 nematodes per plant and incubated under short-day conditions (8 hours light/16 hours dark) at 25 °C. The roots of the seedlings were covered with black paper to mimic the dark underground environment in nature.

### Evaluation of gall formation efficiency

Gall numbers were normalized for each petri dish of six seedlings to evaluate the gall formation efficiency (Data S1). The calculation procedure is as described below: (1) Galls number and germinated seedlings number were counted for each petri dish of six seedlings. (2) Gall number/seedling values were calculated. (3) Average values of galls/seedling in WT were calculated for each independent experiment to define the basal value (highlighted in orange in Data S1). (4) The galls/seedling values from WT and *del1-1* were normalized to the basal value for each petri dish to calculate relative galls number. (5) Average values of relative gall numbers were calculated (highlighted in blue in Data S1). These values were used for Fig. 2A.

### Phloroglucinol-HCl staining

Lignin was detected by the Wiesner test by immersing roots or galls of RKN-infected seedlings in phloroglucinol reagent [1% (w/v) phloroglucinol in 20% (v/v) HCl] for 5 minutes. Stained samples were then mounted in chloral hydrate solution (8 g chloral hydrate, 2 ml ultrapure water and 1 ml glycerol). Samples were imaged with an Axio Imager M1 microscope (Carl Zeiss) mounted with a DP71 digital camera (Olympus).

### Gene expression analysis

Total RNA was extracted from roots or galls (approximately 50 mg fresh weight) using the RNeasy plant mini kit (Qiagen), then treated with Recombinant DNase I (Takara) according to the manufacturer’s instruction. First-strand cDNA was synthesized from 300 ng of total RNA using PrimeScript RT Master Mix (Takara). The transcript level of target genes was assayed using FastStart Essential DNA Green Master (Roche) and the LightCycler 480 system (Roche). The thermal cycler program was 95 °C for 5 min followed by 55 cycles of 95 °C for 10 s, 60 °C for 10 s, and 72 °C for 10 s. *GAPDH* was used as the internal control and relative expression levels were calculated by the ΔΔCt method. Sequences of primers used are listed in Table S1. Each of the three biological replicates were performed in technical triplicates.

### Histological analysis

Galls were dissected and transferred into 2% glutaraldehyde in 20 mM cacodylate buffer, pH 7.4, vacuum-infiltrated for 10 min twice, then incubated in 4 °C overnight. Samples were dehydrated in a graded ethanol series and embedded in Technovit 7100 (Kulzer) according to the manufacturer’s protocol. Sample blocks were sectioned to 5 µm thickness using an ultramicrotome (LEICA RM2255, Leica) and stained with 0.01% (w/v) toluidine blue O (WALDECK) containing 1% (w/v) sodium borate decahydrate (Nacalai) for 2 min. All samples were rinsed in deionized water for 1 min. After drying, sections were mounted in EUKITT (O. Kindler). Samples were imaged with an Axio Imager M1 microscope (Carl Zeiss) mounted with a DP71 digital camera (Olympus). Lengths and areas of the galls were quantified using ImageJ.

### Quantification of total SA

Total SA (free SA plus SA glucosides) from *Arabidopsis thaliana* roots was extracted as previously described^35^. Non-inoculated whole root systems (primary roots and lateral roots) of 12-days-old seedlings, or 7 dpi galls (more than 150 mg fresh weight) were collected. SA of the root exudates was determined by high performance liquid chromatograph–fluorescence detector (HPLC-FL) (LC-2000 plus and FP-2020 plus, Jasco) according to the previous report^35^. Briefly, 20 µL of sample was injected in water/methanol (20:80) eluent to be separated from other organic compounds by using a multi-mode column (Scherzo SM-C18, Imtakt, 2 mm × 50 mm). Fluorescence at 407 nm was monitored with 305 nm excitation for SA and *o*-anisic acid.

### Statistical information

All *P* values were derived from two-sided Student’s *t*-tests or Tukey’s tests. All statistical tests and *n* numbers, including sample sizes or biological replications, are described in the figure legends.

## Supplementary information

Supplementary information.
