# Supplementary material for: The atypical E2F transcription factor DEL1 modulates growth–defense tradeoffs of host plants during root-knot nematode infection
Source: Sci Rep. 2020 Jun 1;10:8836. doi: 10.1038/s41598-020-65733-3 (PMC7264364; doi:10.1038/s41598-020-65733-3)
Supplement: Supplementary file 1 — Supplementary information. [file 41598_2020_65733_MOESM1_ESM.pdf]

The atypical E2F transcription factor DEL1 modulates growth–defense tradeoffs of host plants during root-knot nematode infection

Satoru Nakagami[1], Kentaro Saeki[1], Kei Toda[1], Takashi Ishida[1,2], Shinichiro Sawa[1]*

[1]Graduate School of Science and Technology, Kumamoto University, Kumamoto 860-8555, Japan.

[2]International Research Organization for Advanced Science and Technology (IROAST), Kumamoto University, Kumamoto 860-8555, Japan.

*Corresponding author. Email: sawa@kumamoto-u.ac.jp

Supplemental Information

Supplementary Fig. S1

Supplementary Fig. S2

Supplementary Table S1

Supplementary Data S1

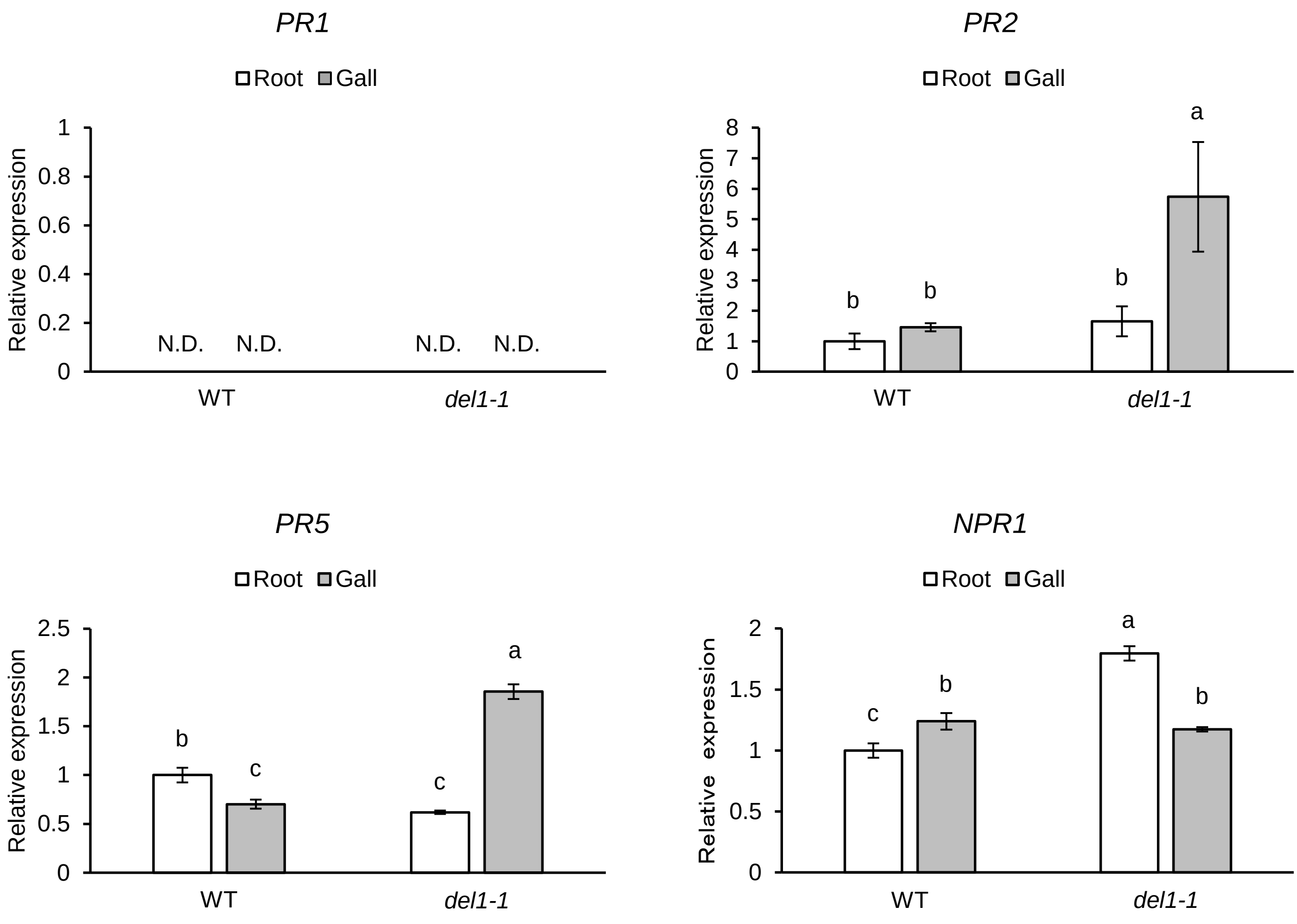

Supplementary Fig. S1. RT-qPCR analysis of *PR1*, *PR2*, *PR5* and *NPR1* in galls of the WT and the *del1-1* mutant. Values are normalized to the expression levels in un-inoculated roots of WT. The experiment was repeated three times with similar results. Means ± SD are shown. Alphabets denote significant difference with Tukey's multiple test at $P < 0.05$. N.D., not detected.

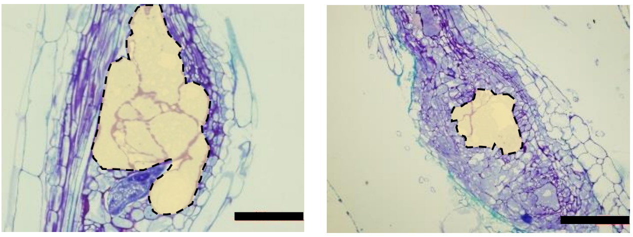

Supplementary Fig. S2. GC formation is compromised in the *del1-1* mutant. Representative WT and *del1-1* 14 dpi gall section images from Figure 1B. Scale bars = 200 µm.

Supplementary Table S1. Oligonucleotides used for RT-qPCR.

Primer name	Sequence
PAL1 qRT-PCR F1	GGTGCTACTTCTCATCGGAGAACC
PAL1 qRT-PCR R1	GAGTGTGGCAATCTGTGGCTT
PAL2 qRT-PCR F1	CTGACAGTTACGGAGTCACCACC
PAL2 qRT-PCR R1	GTTTCCGAATATTCCGGCGTT
C4H qRT-PCR F1	CCTTGAAGCTGAGCAGAAGGG
C4H qRT-PCR R1	TCAGGATGGTTCACTAGCTCTGC
4CL1 qRT-PCR F1	GTGAAATCTGGTGCTGCTCCTC
4CL1 qRT-PCR R1	CCTGCTTCCGTCATTCCGTATC
4CL2 qRT-PCR F1	CCTAACGCCAAGCTTGGTCAG
4CL2 qRT-PCR R1	CCACATGCTCCTGACTTCACTG
CAD5 qRT-PCR F1	GTCAACCTACACAAGGCGGCT
CAD5 qRT-PCR R1	AGTGGCTCAGTGGACTGTACACAG
ICS1 qRT F	GAATTTGCAGTCGGGATCAG
ICS1 qRT R	AATTAATCGCCTGTAGAGATGTTG
EDS5 qRT F	ATCATATCCGAGATGCATAGACTG
EDS5 qRT R	CGAACATTAGTGTGAGACAACCA
PBS3 qRT F	GAGTCATCCGATGTTATATCGG
PBS3 qRT R	GTCCAAGTACTTGTTGTTCCATG
PR1 qRT F	GAAAACTTAGCCTGGGGTAGC
PR1 qRT R	TTCATTAGTATGGCTTCTCGTTCA
PR2 qRT F	CAAGGAGCTTAGCCTCACCA
PR2 qRT R	CGATGGACTTGGCAAGGTATC
PR5 qRT F	CAGTATTCACATTCTCTTCCTCGT
PR5 qRT R	CAATTCAAATCCTCCATCGC
NPR1 qRT F	GAACATCACCGGGTGTAAAGA
NPR1 qRT R	CAGTTCATAATCTGGTCGAGCA

Supplementary Data S1

Definition of control

WT

		No. of galls	/	seedlings	gall/seedling
Experiment 1	Petri dish 1	60	/	6	10.00
	Petri dish 2	58	/	5	11.60
	Petri dish 3	60	/	6	10.00
	Petri dish 4	58	/	5	11.60
				Average	10.80

→ Control value for experiment 1

		No. of galls	/	seedlings	gall/seedling
Experiment 2	Petri dish 5	134	/	6	22.33
	Petri dish 6	97	/	6	16.17
	Petri dish 7	128	/	6	21.33
	Petri dish 8	104	/	6	17.33
				Average	19.29

→ Control value for experiment 2

		No. of galls	/	seedlings	gall/seedling
Experiment 3	Petri dish 9	38	/	6	6.33
	Petri dish 10	27	/	6	4.50
	Petri dish 11	29	/	6	4.83
	Petri dish 12	12	/	6	2.00
	Petri dish 13	42	/	6	7.00
	Petri dish 14	35	/	6	5.83
	Petri dish 15	36	/	5	7.20
				Average	5.39

→ Control value for experiment 3

Calculation

WT		No. of galls	/	seedlings	gall/seedling	relative galls number
Experiment 1	Petri dish 1	60	/	6	10.00	0.93
	Petri dish 2	58	/	5	11.60	1.07
	Petri dish 3	60	/	6	10.00	0.93
	Petri dish 4	58	/	5	11.60	1.07
Experiment 2	Petri dish 5	134	/	6	22.33	1.16
	Petri dish 6	97	/	6	16.17	0.84
	Petri dish 7	128	/	6	21.33	1.11
	Petri dish 8	104	/	6	17.33	0.90
Experiment 3	Petri dish 9	38	/	6	6.33	1.18
	Petri dish 10	27	/	6	4.50	0.84
	Petri dish 11	29	/	6	4.83	0.90
	Petri dish 12	12	/	6	2.00	0.37
	Petri dish 13	42	/	6	7.00	1.30
	Petri dish 14	35	/	6	5.83	1.08
	Petri dish 15	36	/	5	7.20	1.34
					Average	1.00

del1-1		No. of galls	/	seedlings	gall/seedling	relative galls number
Experiment 1	Petri dish 1	67	/	6	11.17	1.03
	Petri dish 2	35	/	5	7.00	0.65
	Petri dish 3	35	/	5	7.00	0.65
	Petri dish 4	16	/	6	2.67	0.25
Experiment 2	Petri dish 5	87	/	6	14.50	0.75
	Petri dish 6	118	/	6	19.67	1.02
	Petri dish 7	81	/	6	13.50	0.70
	Petri dish 8	65	/	6	10.83	0.56
	Petri dish 9	88	/	6	14.67	0.76
Experiment 3	Petri dish 10	17	/	5	3.40	0.63
	Petri dish 11	16	/	5	3.20	0.59
	Petri dish 12	25	/	6	4.17	0.77
	Petri dish 13	20	/	6	3.33	0.62
	Petri dish 14	13	/	5	2.60	0.48
	Petri dish 15	12	/	6	2.00	0.37
	Petri dish 16	10	/	6	1.67	0.31
					Average	0.63

Calculation procedure

1. Galls number and germinated seedlings number were counted for each petri dish.
2. Gall/seedling values were calculated.
3. Average values of gall/seedling in WT were calculated for each independent experiment to define control value (highlighted in orange).
4. Gall/seedling values in the WT and *del1-1* were normalized by each control value for each petri dish to calculate relative galls number.
5. Average values of relative galls numbers were calculated (highlighted in blue), and were used for drawing Figure 2A.
